# Respiratory symptoms and ventilatory functions among quarry workers in Edo state, Nigeria

**DOI:** 10.11604/pamj.2016.23.212.7640

**Published:** 2016-04-21

**Authors:** Alphonsus Rukevwe Isara, Vincent Yakubu Adam, Adesuwa Queen Aigbokhaode, Innocent Osi Alenoghena

**Affiliations:** 1 Department of Community Health, University of Benin Teaching Hospital, P. M. B. 1111, Benin City, Nigeria

**Keywords:** Lung function, quarry workers, respiratory symptoms, Nigeria

## Abstract

**Introduction:**

Workers in the quarry industries are exposed to hazards resulting from the inhalation of air borne particulates. The study determined the prevalence of respiratory symptoms and assessed ventilatory functions among quarry workers in Edo state, Nigeria.

**Methods:**

Quarry workers (site workers and office workers) were interviewed using structured questionnaire. FEV1, FVC, FEV1/FVC and PEFR were measured using a KoKo Legend spirometer.

**Results:**

A total of 113 quarry workers (76 exposure and 37 controls) were studied. The exposure group had significantly higher occurrence of chest tightness (35.5%) compared with 16.2% of the controls (p < 0.05). The occurrence of cough (23.7% versus 13.5%), sputum (21.1% versus 16.2%), and dyspnoea (7.9% versus 5.4%), were higher in exposure groups while wheeze (10.8% versus 10.5%) and nasal congestion (27.0% and 25.0%) were higher in the control groups. The mean (SD) FEV1, and FVC were significantly lower among the exposure compared with the control group; 2.77L (0.73) versus 3.14L (0.78), p < 0.05, and 3.48L (0.84) versus 3.89L (0.92), p < 0.05. In both groups, smokers had significantly lower mean (SD) FEV1, FVC and PEFR compared with non-smokers; 2.91L (0.77) versus 3.39L (0.69), p = 0.01, 3.61L (0.91) versus 4.26L (0.74), p < 0.05 and 6.56L (2.43) versus 7.98L (1.67), p < 0.05.

**Conclusion:**

Chronic exposure to quarry dust is associated with respiratory symptoms and reduced lung function indices among quarry workers. The enforcement of the use of PPEs and periodic evaluation the lung function status of quarry workers is advocated.

## Introduction

The art of quarrying is an age long economic activity in areas endowed with natural resources such as marble, lime stone and gypsum. Workers in the quarry industries are exposed to various hazards resulting from the inhalation of air borne particulates and these poses a lot of danger to their health and safety. Airborne particulates pose a potential health risk to quarry employees in the form of respiratory, dermal, ocular irritation and damage [1–4]. Chronic exposure to dust generated from crushing of granite rocks impairs lung function and causes some respiratory and non-respiratory symptoms in quarry workers [1, 2]. A particular concern in some quarries is the inhalation of dust containing silica which can lead to silicosis, an irreversible lung disease resulting in inflammation of the lungs and breathing difficulties which progresses even when exposure stops [5, 6]. Reports from countries like the United States and China have shown that millions of people exposed to silica dust eventually develop silicosis resulting in many deaths especially among the older workers [7]. In many developing countries of the world especially in Africa, health and safety practices which are a systematic ways to control all possible hazards that might lead to unsafe conditions for employees of an industry are not well established [6]. In Nigeria there is still paucity of literature on health and safety measures among quarry workers as the few studies carried out were in the Eastern and Northern part of Nigeria [6, 8]. A preliminary study among quarry workers in Edo State, in southern Nigeria, showed a dearth of awareness of the hazards and diseases associated with working in the quarry industry and thus poor use of safety equipments [3]. Our hypothesis was that exposure to quarry dust is associated with respiratory symptoms and abnormal ventilatory functions. Therefore, this study described the burden of respiratory symptoms and abnormal ventilatory functions arising from exposures to quarry dust among the quarry workers in Edo State, Nigeria. It also assessed the risk factors associated with abnormal ventilatory function among quarry workers. It also related the duration of exposure to quarry dust in the quarry environment to the manifestation of lung function abnormalities. Thus the burden of lung function disorders was estimated for the first time in Edo State, Nigeria.

## Methods

**Study setting:** This descriptive cross-sectional study was carried out in Ikpeshi community, a rural settlement located in Akoko-Edo local government area of Edo State, in the southern region of Nigeria. The community which is inhabited by mostly indigenous farmers, migrant traders and quarry workers is bounded in the East by Auchi, in the West by Igarra, in the North by Uneme-Nikwa and in the South by Ihiezbe-Ogbe. It is a mountainous area richly endowed with immense deposits of mineral stones and rocks making it a place for sourcing, quarrying and mining of different type of stones. The quarries which are owned by individuals or private organizations comprises of mountainous rocks occupying a wide expanse of area in the interior part of the community.

**Study population:** This study population comprised of quarry workers working in the quarries both in the factories where the actual activities of mining, milling, blasting, breaking, bagging and loading of stones takes place and in the offices where administrative activities takes place. Adult quarry workers aged 18 years and above who have worked in the quarry for at least one year were recruited for the study. A minimum of one year working duration was taken to enable us ascertain that the worker(s) have been adequately exposed to quarry dust. Quarry workers aged below 18 years and those with contraindication to spirometry (recent or current pneumothorax, hemoptysis, recent abdominal or eye surgery, unstable cardiovascular status e.g. aneurysm, recent heart attack) were excluded from the study. The quarry workers were divided into two groups based on the nature of their job in the quarries. Workers in the production area who are involved in milling, grinding, breaking and loading of stones were designated the as “site workers” or exposure while those who work in the administrative offices were designated the “office workers” or control groups. Quarry workers who met the inclusion and gave informed consent were recruited consecutively from the quarries according to their respective sizes to participate in the study.

**Data collection:** The instruments for data collection were a questionnaire and the Koko Legend Spirometer (Model 314000, Serial 2007LB0538). The questionnaire was adapted from relevant sections of the Burden of Obstructive Lung Diseases (BOLD) questionnaire. It was used to collect information on the socio-demographic characteristics and respiratory symptoms of the study participants. The respiratory symptoms of interest were cough, sputum, wheezing, chest pain, dyspnoea and nasal congestion. The questionnaire was administered on the study participants by the researchers. In preparation for Spirometry, the environment was monitored and ambient conditions within accepted ranges. Trained persons conducted spirometry. Calibration of the spirometer was performed daily before the first participant blows into it and after the 8th participant or after 4 hours of continuous usage. All calibration was done using a valid certified accurate 3L calibration syringe. In the Spirometry, a minimum of 3 and a maximum of 8 acceptable and repeatable forced expiratory manoeuvres were done with the study participant seated and upright according to ATS/ERS 2005 criteria. The best test was the one used for interpretation. This is the test from an acceptable curve that has the highest sum of forced vital capacity (FVC) and forced expiratory volume in one second (FEV1) into the expiratory maneuver. Each test was signed by the operator, dated and comments made on the patient's effort and technique.

**Data analysis:** an initial univariate analysis was conducted for all variables to assess the distribution for each variable to establish whether they are within acceptable range. Categorical variables were summarized using proportions. Continuous variables were summarized using means and standard deviations. The FEV1, FVC, FEV1/FVC, peak expiratory flow rate (PEFR) and the respiratory symptoms were the primary outcome variables. Airflow obstruction was defined as FEV1/FVC <70%. The socio-demographic characteristics and duration of exposure to quarry dust constituted the independent variables while smoking and indoor cooking were the secondary outcome variables. The student's t test was used to compare means while the chi square test was used to test for associations. The level of significance was set at a p value of less than 0.05.

**Ethical approval:** ethical approval was granted by the University of Benin Teaching Hospital Ethics and Research Committee. Institutional consent was obtained from the management of the respective quarries while a written informed consent was obtained from the quarry workers after a detailed explanation of the purpose of the study.

## Results

A total of 113 quarry workers comprising 76 site workers and 37 office workers were studied. Table 1 shows the socio-demographic and other characteristics of the respondents. The controls with a mean age of 42.6 ± 12.7 years were significantly older than the exposure groups with a mean age of 36.2 ± 10.9 years (p < 0.05) and a higher proportion of them were married (p < 0.05). Both groups were similar in other demographic characteristics. A higher proportion of both the exposure and control groups have worked in the quarries for 1 - 5 years, 41 (53.9%) and 14 (37.8%) respectively. Less than quarter of the respondents in both groups were smokers. Indoor cooking was practiced by 43 (43.4%) of site workers and 20 (54.1%) of office workers. Kerosene (73.7% for exposures and 89.2% for controls) and firewood (61.8% for exposures and 54.1% for controls) were the predominant types of fuel used for cooking by the respondents. Other types of cooking fuel used were cooking gas (3.9% for exposures and 10.8% for controls) and electricity (9.2% for exposures and 13.5% for controls). The exposure group had significantly higher occurrence of chest tightness (35.5%) compared with 16.2% of the controls (p < 0.05). The occurrence of cough (23.7% versus 13.5%), sputum (21.1% versus 16.2%), and dyspnoea (7.9% versus 5.4%), were higher in exposure groups while wheeze (10.8% versus 10.5%) and nasal congestion (27.0% and 25.0%) were higher in the control groups. However, these differences were not statistically significant (Table 2). Table 3 shows the lung function parameters of the respondents. The mean (SD) FEV1, and FVC were significantly lower among the exposure compared with the control group; 2.77L (0.73) versus 3.14L (0.78), p = 0.02, and 3.48L (0.84) versus 3.89L (0.92), p < 0.05. In both groups, smokers had significantly lower mean (SD) FEV1, FVC and PEFR compared with non-smokers; 2.91L (0.77) versus 3.39L (0.69), p = 0.01, 3.61L (0.91) versus 4.26L (0.74), p < 0.05 and 6.56L (2.43) versus 7.98L (1.67), p < 0.05. There was no statistically significant difference in the lung function parameters of both groups irrespective of whether they cooked indoors or not. The factors associated with respiratory symptoms among the respondents are shown in Table 4. All the respiratory symptoms assessed in this study were higher among respondents who have worked 1 - 5 years in the quarries. Cough, wheeze and nasal congestion were more prevalent among respondents who cook indoors while all the respiratory symptoms were lower among smokers than non smokers. Work duration and indoor cooking were not statistically associated with respiratory symptoms among the quarry workers.

**Table 1:** Socio-demographic and other characteristics of respondents

Variables	Exposure n (%)	Control n (%)	P-value
Mean age; years (SD)	36.2 (10.9)	42.6 (12.7)	**0.01**
Mean weight; kg (SD)	65.6 (9.4)	67.1 (11.3)	0.46
Mean height; m (SD)	1.71 (0.1)	1.71 (0.1)	0.85
**Sex**			
Male	56 (73.7)	29 (78.4)	0.59
Female	20 (26.3)	8 (21.6)	
**Marital status**			
Married	52 (68.4)	33 (89.2)	**0.02**
Single	24 (31.6)	4 (10.8)	
**Educational status**			
Primary	22 (28.9)	8 (21.6)	0.25
Secondary	43 (56.6)	19 (51.4)	
Tertiary	11 (14.5)	10 (27.0)	
**Duration of work (years)**			
1 – 5	41 (53.9)	14 (37.8)	0.18
6 – 10	21 (27.6)	11 (29.7)	
> 10	14 (18.4)	12 (32.4)	
**Smoking**			
Yes	18 (23.7)	8 (21.6)	0.81
No	58 (76.3)	29 (78.4)	
**Indoor cooking**			
Yes	33 (43.4)	20 (54.1)	0.29
No	43 (56.6)	17 (45.9)	

**Table 2:** Prevalence of respiratory symptoms among the quarry workers

Symptoms	Exposure n (%)	Control n (%)	P-value
**Cough**			
Yes	18 (23.7)	5 (13.5)	0.21
No	58 (76.3)	32 (86.5)	
**Sputum**			
Yes	16 (21.1)	6 (16.2)	0.54
No	60 (78.9)	31 (82.8)	
**Dyspnoea**			
Yes	6 (7.9)	2 (5.4)	0.63
No	70 (92.1)	35 (94.6)	
**Wheeze**			
Yes	8 (10.5)	4 (10.8)	0.96
No	68 (89.5)	33 (89.2)	
**Chest pain**			
Yes	27 (35.5)	6 (16.2)	0.03
No	49 (64.5)	31 (83.8)	
**Nasal congestion**			
Yes	19 (25.0)	10 (27.0)	0.85
No	57 (75.0)	27 (73.0)	

**Table 3:** Mean (SD) values of lung function results of the quarry workers

Parameters	Exposure	Control	P-value	Smoking		P-value	Indoor cooking		P-value
				Yes	No		Yes	No	
FEV1 (L)	2.77 (0.73)	3.14 (0.78)	0.02	2.91 (0.77)	3.39 (0.69)	0.01	3.06 (0.75)	2.99 (0.81)	0.67
FVC (L)	3.48 (0.84)	3.89 (0.92)	0.03	3.61 (0.91)	4.26 (0.74)	0.01	3.75 (0.86)	3.76 (0.96)	0.93
FEV1/FVC	0.79 (0.09)	0.81 (0.07)	0.53	0.81 (0.08)	0.79 (0.07)	0.46	0.81 (0.07)	0.79 (0.08)	0.26
PEFR (L/s)	6.66 (2.48)	6.99 (2.29)	0.49	6.56 (2.43)	7.98 (1.67)	0.01	6.99 (2.35)	6.78 (2.36)	0.63

FEV1 = Forced expiratory volume in one seconds; FVC = forced vital capacity; PEFR = peak expiratory flow rate

**Table 4:** Factors associated with respiratory symptoms of the quarry workers

Variables	Cough	Sputum	Dyspnoea	Wheeze	Chest pain	Nasal congestion
**Duration of work**						
1 – 5 years	11 (47.8)	14 (63.6)	6 (75.0)	9 (75.0)	17 (51.5)	13 (44.8)
6 – 10 years	7 (30.4)	3 (13.6)	0 (0.0)	3 (25.0)	12 (36.4)	7 (24.1)
> 10 years	5 (21.7)	5 (22.7)	2 (25.0)	0 (0.0)	4 (12.1)	9 (31.1)
P value	0.96	0.18	0.19	0.08	0.17	0.48
**Indoor cooking**						
Yes	13 (56.5)	10 (45.5)	4 (50.0)	7 (58.3)	15 (45.5)	17 (58.6)
No	10 (43.5)	12 (54.5)	4 (50.0)	5 (41.7)	18 (54.5)	12 (41.4)
P value	0.30	0.88	0.86	0.40	0.84	0.14
**Smoking**						
Yes	4 (17.4)	4 (18.2)	1 (12.5)	3 (25.0)	4 (12.1)	6 (20.7)
No	19 (82.6)	18 (81.8)	7 (87.5)	9 (75.0)	29 (87.9)	23 (79.3)
P value	0.47	0.55	0.46	0.86	0.07	0.73

## Discussion

This study showed that the prevalence of respiratory symptoms was remarkably higher among quarry workers who work in the sites compared to their counterparts working in the offices. It can be deduced that the site workers are exposed to higher levels of quarry dust. The limited and non use of personal protected equipment (PPE) by quarry workers which has been documented previously in the study locale [3] may have also contributed this high prevalence of respiratory symptoms. Nwibo et al reported a higher prevalence of 47.6% for cough and 40.7% for chest pain among quarry workers in Ebonyi state, south eastern Nigeria [9]. Also reports from studies among quarry workers in Brazil [10] showed a prevalence of irritant cough of 57.1% while in Iran [11], a prevalence of cough of 31.9% was recorded. In the Ebonyi state study, non use of PPEs was found among 98.3% of the quarry workers studied and this is comparable to the situation in our study.

In this study, a higher prevalence was recorded for all the respiratory symptoms among quarry workers who have worked for duration of 1 - 5 years in the quarries. Although this association was not statistically significant, this finding contrasted a study in India which showed that exposure duration of more than 15 years was associated with silicosis and many more respiratory problems among sand stone quarry workers [12]. Our findings may be explained by the fact that quarry workers who have worked for a longer duration may be too ill to continue working in the quarries as almost half of the respondents in this study have worked for 1 - 5 years (healthy worker effect). Also, it could be that the workers who have worked for a longer duration in the quarries would have developed tolerance to the effect of quarry dust over time. It is also possible for workers to conceal their symptoms so that they can continue work in the quarries. The prevalence of cough, wheeze and nasal congestion was higher among quarry workers who cook indoors. This association was not statistically significant despite the fact that majority of them used unclean cooking fuel such as firewood and kerosene. This may be attributed to most of the quarry workers being males who probably spend long hours in quarries and spending less time at home. Also majority of the respondents were married in this study. This indicates that they are not primarily responsible for cooking as it is culturally the duty of women to cook for other members of the family in our environment. Thus, younger persons who are unmarried will be more exposed to indoor air pollution resulting from cooking activities. It has been documented that exposure to indoor air pollution is associated with acute and chronic respiratory disorders [13–15]. Smoking was not significantly associated with the prevalence of respiratory symptoms in this study. The relatively few smokers identified among the quarry workers may be responsible for this finding. The mean values of FEV1 and FVC of the site workers was significantly lower than those of the office workers. This was consistent with findings among men and women exposed to dust generated from crushing granite rocks in Calabar, Nigeria [1] and the Ebonyi state study [9].

A similar result was reported in two studies in India [16, 17]. Although we did not carry out dust sampling of the site and office environment in this study, it is expected that the level of respirable dust will be higher in the site environment than the office environment. Thus prolonged exposure to quarry dust coupled with non use of PPEs will ultimately lead to deterioration of lung function indices. The mean values of FEV1, FVC and PEFR were significantly lower among the quarry workers who smoke cigarette. Again this is consistent with many previous studies [9, 17] but in contrast to this finding, Gupta et al observed no difference in the lung function results of quarry workers who are smokers and non smokers in Rajasthan, India [16].

A limitation of this study is that it relied on information from the study participants to estimate the respiratory symptoms. Therefore it may be prone to recall and information bias. Also, dust sampling would have made it possible to quantify and compare the concentration of respirable dust in both the site and office environments of the quarries.

## Conclusion

This study showed that respiratory symptoms were more prevalent among workers who had chronic exposure to quarry dust and those who smoke cigarette. Also, chronic exposure to quarry dust was associated with deterioration of lung function indicated by reduced lung function indices. The result of this study will serve as an advocacy tool that will drive the government authorities on the need for legislature that will mandate quarry owners to provide PPEs such as respirators and facemask for their workers. The enforcement of the use of PPEs and periodic evaluation the lung function status of quarry workers is also advocated.

### What is known about this topic

- Workers in the quarry industries are exposed to various hazards resulting from the inhalation of air borne particulates;
- Chronic exposure to quarry dust is associated with higher prevalence of respiratory symptoms and impaired lung functions among quarry workers;
- Low level of awareness of hazards associated with working in the quarry and poor use of safety equipment by quarry workers.

### What this study adds

- Workers in the site environment of quarries have lower lung function parameters compared to those in the office environment. This may have resulted from a higher level of quarry dust exposures;
- The need to ensure the enforcement of the use of PPEs and periodic evaluation the lung function status of quarry workers.
